# Capacity building through comprehensive implementation research training and mentorship: an approach for translating knowledge into practice

**DOI:** 10.1186/s12992-023-00935-8

**Published:** 2023-05-25

**Authors:** Emmanuel Asampong, Edward Mberu Kamau, Philip Teg-Nefaah Tabong, Franklin Glozah, Adanna Nwameme, Kwabena Opoku-Mensah, Belynda Amankwa, Phyllis Dako-Gyeke

**Affiliations:** 1 Department of Social and Behavioural Sciences, School of Public Health, College of Health Sciences, University of Ghana, Legon, Ghana; 2 UNICEF/UNDP/World Bank/WHO, The Special Programme for Research and Training in Tropical Diseases (TDR), World Health Organization, Geneva, Switzerland; 3 United Nations Development Programme, Ghana Office, Accra, Ghana

**Keywords:** Implementation research, Capacity strengthening, Mentorship, LMICs, Public health

## Abstract

**Background:**

Implementation research (IR) is increasingly gaining popularity as the act of carrying an intention into effect. It is thus an important approach to addressing individual practices, policies, programmes and other technologies to solving public health problems. Low- and middle-income countries (LMICs) continue to experience public health problems which could be addressed using implementation research. These countries however fall behind prioritizing implementation research due to the disorganized approach used to providing knowledge about the value and scope of implementation research. This paper seeks to explain steps taken to resolve this by capacity strengthening activities through a comprehensive implementation research training and mentorship programme which was informed by needs assessment.

**Methods:**

The roll-out of the comprehensive implementation research training and mentorship was done in phases, including engaging the implementation research community through TDR Global, competency building for programme officers and ethical review board/committee members, and practical guidance to develop an implementation research proposal. The Bloom taxonomy guided the training whilst the Kirkpatrick Model was used for the evaluation of the effectiveness of the capacity building.

**Results:**

The findings identified critical areas of mentors and how mentorship should be structured and the most effective ways of delivering mentorship. These findings were used to develop a mentorship guide in IR. The mentorship guidance is to be used as a check-tool for mentoring participants during trainings as part of the package of resources in implementation research. It is also to be used in equipping review board members with knowledge on ethical issues in implementation research.

**Conclusion:**

The approach for providing comprehensive implementation research training and mentorship for programme personnel has provided an opportunity for both potential mentors and mentees to make inputs into developing a mentorship guidance for LMICs. This guidance would help address mentorship initiation and implementation challenges in IR.

**Supplementary Information:**

The online version contains supplementary material available at 10.1186/s12992-023-00935-8.

## Background

Implementation research (IR) has become a major discipline that is crucial in addressing problems encountered when implementing health interventions that have been proven to be efficacious. Consequently, employing IR generates improved knowledge required to address significant questions faced by health programme implementers, health practitioners, policy makers and communities on best ways in facilitating and strengthening implementation, effectiveness, efficiency, sustainability, and fidelity of interventions known to improve individual and population health [1]. The challenges associated with the know-do gap in real world settings and the contextual issues pertaining to specific countries are thus addressed through IR to yield positive national and global health outcomes [2].

To ensure that this happens, several capacity-strengthening initiatives have been rolled out over the years to provide the requisite competencies for intervening against disease conditions of public health importance [3–6]. Besides, with the emergence of disease agents such as Severe Acute Respiratory Syndrome Coronavirus 2 (SARS-CoV 2) which causes COVID-19, it has become more imperative that there is efficient generation, management, and dissemination of public health information to effectively intervene against malaria, Neglected Tropical Diseases (NTDs), tuberculosis (TB), and other infections especially in Low- and Middle-Income Countries (LMICs) such as Ghana, where the disease burden is high. In respect of malaria for example, even though Ghana has made notable improvement in its fight, where cases and deaths have reduced over 50% and 65% between 2005 and 2015 [7], the burden of malaria remains unacceptably high accounting for 30% of outpatient attendances and 23% inpatient admissions [8].

NTDs, the group of preventable and treatable yet neglected diseases affect 1.5 billion people globally, 40% of whom live in Africa [9]. To achieve elimination of NTDs in Ghana, the Neglected Tropical Diseases Programme (NTDP) of Ghana was introduced in 2006 with a national level office to provide oversight over activities implemented at regional and district levels. This was to largely depend on the health system from regional to district levels. Even though implementation has been relatively successful, some objectives of the programme are yet to be realized [10]. Failure to realise all the objectives has been attributed to the complex systems within the policy context [11]. In relation to TB, a recent survey reported a prevalence of smear-positive TB of 111 per 100,000 among adult population, and prevalence of bacteriologically confirmed TB was 356 per 100,000 population [12]. Several studies have also reported the emergence of Multiple Drug Resistant (MDR)TB in Ghana [13–18].

From the foregoing indices, it is important that IR is given the desired priority, taking cognizance of the contextual and health system peculiarities in Ghana [19] and to address bottlenecks in implementation. This will help quicken the pace of progress at all fronts where there are problems. Currently, that has not been fully realized because there is lack of adequate knowledge about the value and scope of implementation research, a bottleneck which can be resolved by a package of Comprehensive Implementation Research Training and Mentorship (CIRT-M) activities, that recognises that successful scale up of evidence-based interventions into practice relies on contextual factors [20]. In Ghana, most programmes including malaria, NTDs, and TB are implemented in the districts. It is therefore important that IR training is targeted at the district programme officers. Many of these officers lack the requisite competencies in IR. Training needs assessment was nested into a Massive Open Online Courses (MOOC) on IR, which preceded the roll out of this mentorship programme. Besides, they do not get frequent exposure to capacity building efforts [21]. For this to be addressed, a training model that initiates mentoring for programme officers who may lack research experiences becomes imperative [21]. This approach offers a dynamic learning opportunity for the acquisition and sharing of knowledge for both mentors and mentees [22]. This paper reports on a structured approach that piloted a training model for diverse participants (researchers, ethical review board members, mentees, and mentors) within the research value chain to acquire the needed skills to enable them to play their respective roles.

## Conceptual framework for capacity building

The capacity building programme was guided by Bloom’s taxonomy. Bloom identified six levels within the cognitive domain, from the simple remembering of facts, as the lowest level, through to a more complex level, creating [23]. During the capacity building, more emphasis was placed on the creating component of Bloom’s hierarchy. As such, participants were made to translate the knowledge acquired after each session into developing a capstone (research proposal in IR). These were assessed and used as one of the requirements for the award of a certificate. We also adopted the Kirkpatrick levels Model [24] for evaluation of the effectiveness of capacity building and mentorship programme. Kirkpatrick model proposes four level of evaluation of training programmes: reaction, learning, behaviour and result [25]. To start with, all participants in the training were made to respond to a structured questionnaire covering knowledge on IR before the training, which served as baseline data. Post training evaluations were carried out and compared with baseline to measure the extent to which participants acquired knowledge (learning). Participants were also made to complete a training evaluation form to determine the relevance of the training to the job as well as their satisfaction (reaction). After the training, participants were advised to identify IR problems at their place of work and to develop a research proposal to address the implementation challenges of that intervention. These proposals were assessed and selected proposals were provided with funding support to conduct the research. This was done to evaluate the behaviour and result component of the capacity building as suggested by Kirkpatrick [25].

## Methods and activities

**Fig. 1:**
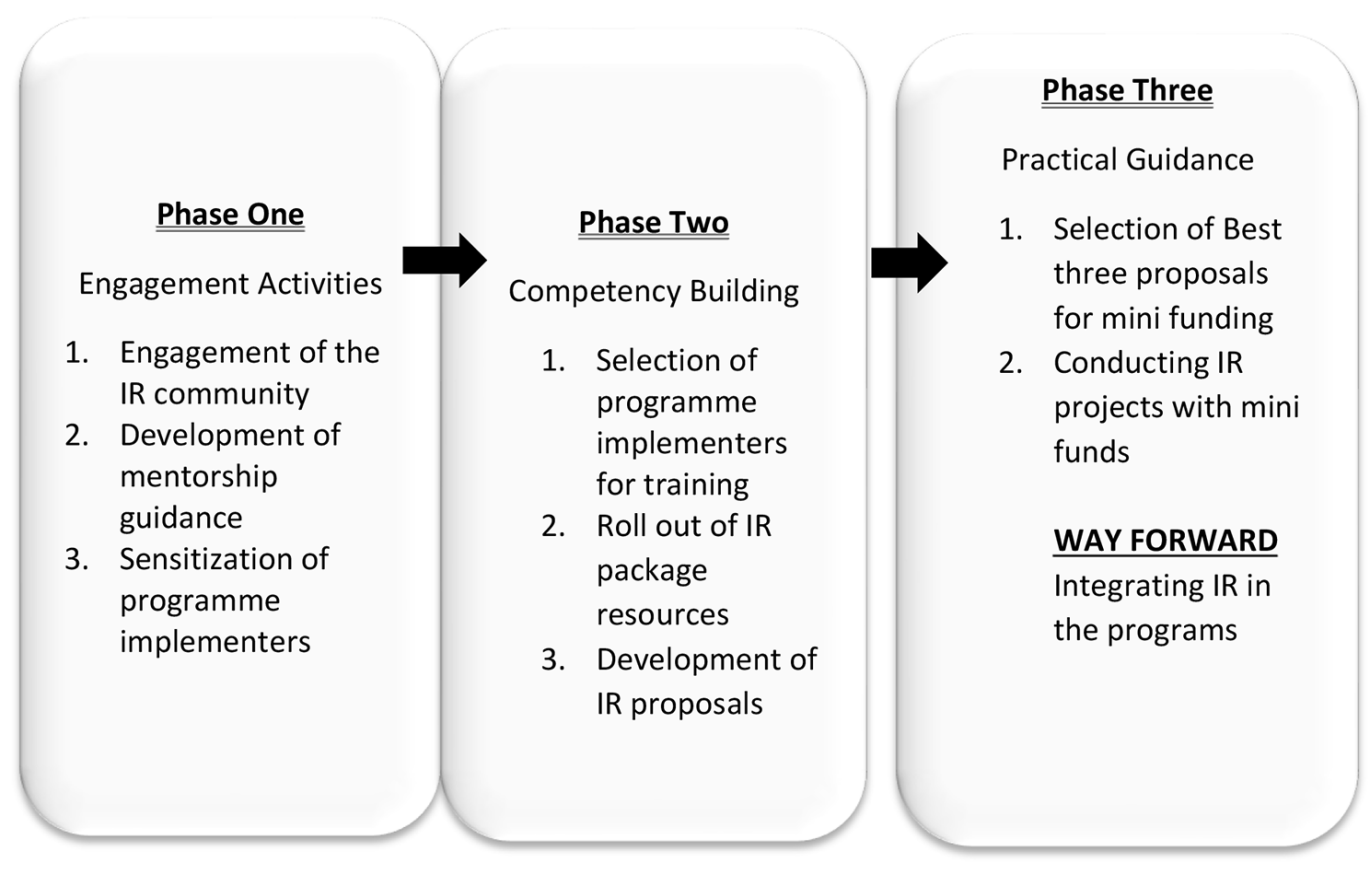
The three-phase approach adopted in this project

**Fig. 2:**
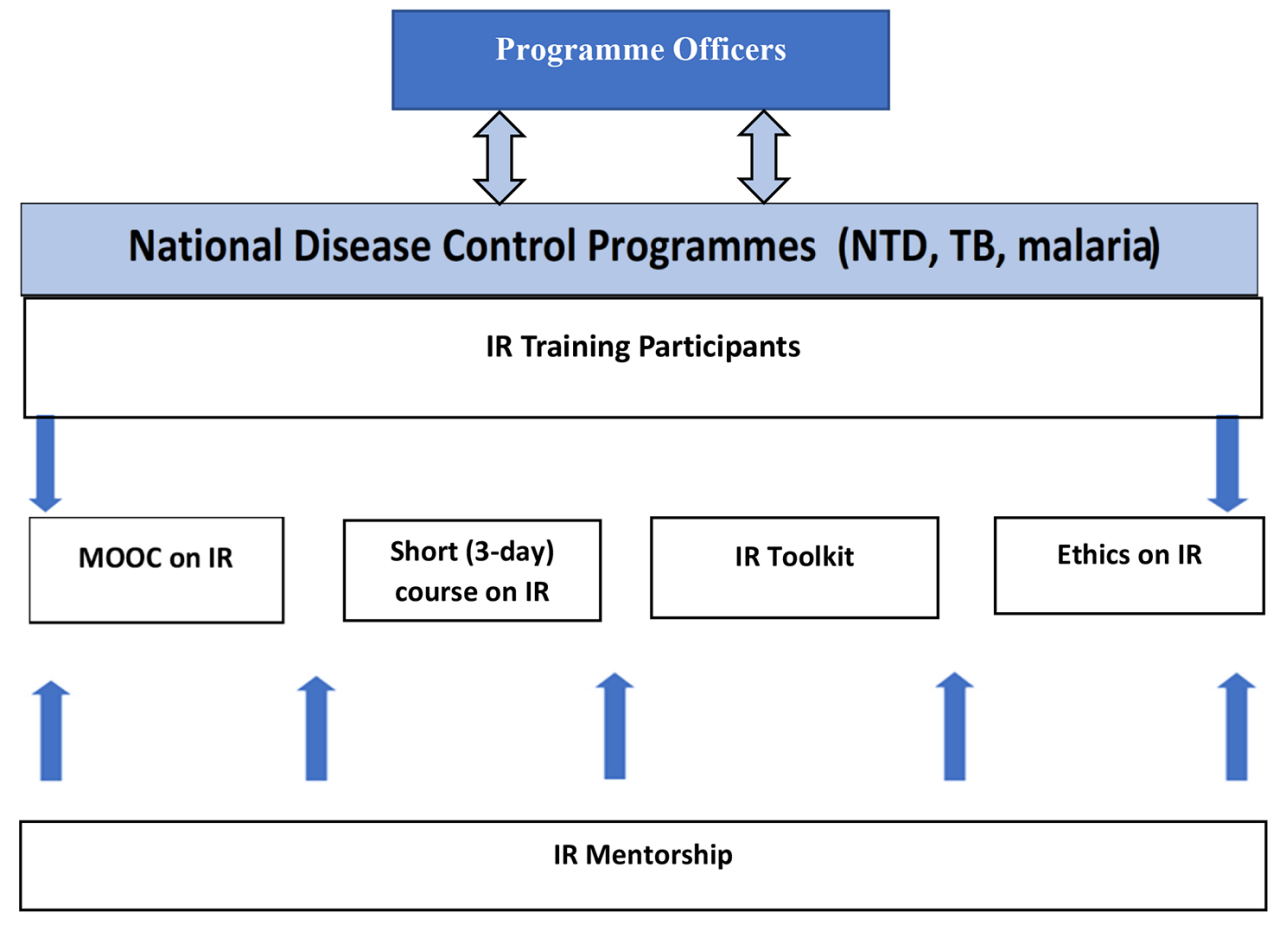
IR resources used for the training

In rolling out the activities, a three-phase approach was adopted (Fig. 1) to provide training on all the IR package training resources (Fig. 2).

### Phase one: engaging the Ghanaian IR community within TDR global

TDR Global is a worldwide community of passionate scientists and experts who have been working with TDR on research on infectious diseases of poverty. Everyone in this community comes with unique knowledge, and together make up a vibrant scientific community. This group of scientists are committed to driving and encouraging mentoring of young scientists and fostering research collaborations. Among them are Ghanaian scientists with rich experiences associated with diseases of poverty, and who are willing to cconnect, network, share experiences and try new methodologies to foster collaboration among people associated with TDR. Besides, they are abreast with the context and health system dynamics of the country. Upon establishing contact with the IR community, we drew on that pool of indigenous scientists who are conversant with IR processes to identify potential and interested mentors.

Identified mentors and mentees participated in series of workshops to develop mentorship guidance to serve as a blue-print document that stipulates the roles, responsibilities and expectations of mentees and mentors. The participation of mentors in these workshops emphasized their significant place as stakeholders in IR. Some of these mentors were members of ethical review committees/boards who required an orientation on ethical issues in IR. Consequently, representatives of ethical review committees/boards within academia and the Ghana Health Service were nominated by their respective committees/boards to participate in the training in ethical issues in IR.

The Ghana Health Service is organized into a three-tiered administrative system: National, Regional and District levels but is five-tiered in terms of service delivery: National, Regional, District, Sub-district and Community Health Planning and Services (CHPS) Zones. For all these levels, there are programme implementers who are required to provide leadership for the health sector response to fighting diseases in their respective jurisdiction. In essence, these personnel ensure that practices, policies, programmes, and other technologies, which are collectively called interventions, yield the desired outcomes. A sensitization programme on IR was undertaken for key programme implementers who are responsible for public health problems particularly malaria, Neglected Tropical Diseases (NTDs), tuberculosis (TB) and infections due to emerging disease agents’ such as COVID-19 at all these levels. During the sensitization activities, the range of CIRT-M activities (Massive Open Online Course-MOOC on IR, Short 3-day course on IR, IR Toolkit and Ethics on IR) was publicized to the programme implementers.

### Phase two: competency building

To ensure that only committed programme implementers were engaged, they were informed to first enroll and participate in the MOOC on IR, an approximately three-month period course that includes quizzes, final exam, and peer assessments. Subsequently, a selection of participants who satisfied all the requirements of the MOOC were identified and notified to participate in a three-day short course on Principles in Implementation Research (PIR), developed by the African Regional Training Centre (ARTC) with support from the WHO/TDR. The content of this course is shown in Table 1.

**Table 1:** Modules and Units in PIR

MODULE 1: Introduction to IR		
Unit 1	Concepts in IR	
Unit 2	Scope of IR	
Unit 3	Phases of IR	
Unit 4	Application and Review	
**MODULE 2: Stakeholder Engagement in IR**		
Unit 1	Community Entry in IR	
Unit 2	Community Engagement in IR	
Unit 3	Stakeholder Analysis	
Unit 4	Dissemination and Scale-up in IR	
**MODULE 3: Methodology in IR**		
Unit 1		Formulating IR problems
Unit 2		Research Approaches
Unit 3		Qualitative Methods
Unit 4		Mixed Methods
Unit 5		Module Summary
**MODULE 4: Ethics in IR**		
Unit 1		Ethics, Principles, History, Guidelines
Unit 2		Ethical Considerations in IR
Unit 3		Ethics review and Informed Consent
Unit 4		Quality Management

Additionally, participants were exposed to the online IR toolkit (https://www.adphealth.org/irtoolkit/), which is designed to help in the conduct of an IR project through a standard process to ensure that high quality results that are reliable are yielded. The participants through an online google form provided information on their training needs and understanding of mentorship.

Ethical review committee/board members were also provided training that included five key sessions as shown in Table 2.

**Table 2:** Content of Ethical Issues in IR

Ethical Issues in IR
1. Introduction to IR
2. Ethical Issues in IR
3. Ethical Issues in Planning Phase of IR
4. Ethical Issues in Implementation Phase of IR
5. Ethical issues in post-Implementation Phase of IR

## Phase three: practical guidance

Upon completion of the training activities, participants were expected to translate the knowledge gained from the range of IR package resources into developing proposals aimed at addressing an IR problem in their programme area. This was done with active guidance and support from mentors who had been assigned to them. Proposals received were sent to three reviewers and at the end, three proposals that scored highest were selected for small project grant funding to conduct proposed studies. The conduct of the studies would culminate into real life IR project that would yield findings to improve programme implementation and scale-up. It would also afford hands-on experience to these programme implementers who would continuously receive guidance from their mentors. It is expected that upon completion of the projects, comprehensive learnings would have taken place such that participants subsequently can solely or with minimal guidance develop IR proposals, seek funding, and conduct IR projects successfully.

## Results

The CIRT-M was initiated to provide skills in translating IR knowledge through to developing proposals addressing IR problems and engaging actively in the conduct of IR projects. The results present demographic characteristics of participants as well as their views on the useful of CIRT-M.

### Demographic characteristics of participants

A total of two hundred and seventy-one (271) participants of whom one hundred and twenty-three (123) were programme implementers, all from the Ghana Health Service enrolled and participated in the MOOC. Of the 271 participants, 150 (55.2%) were males, 120 (44.4%) were females whilst 1 (0.4%) did not want to disclose their sex. One hundred and seventeen 117 (43%) had bachelor’s degree, 114 (42.2%) had master’s degree whilst 5 (1.8%) had doctorate.

### Survey on areas of mentorship

**Fig. 3:**
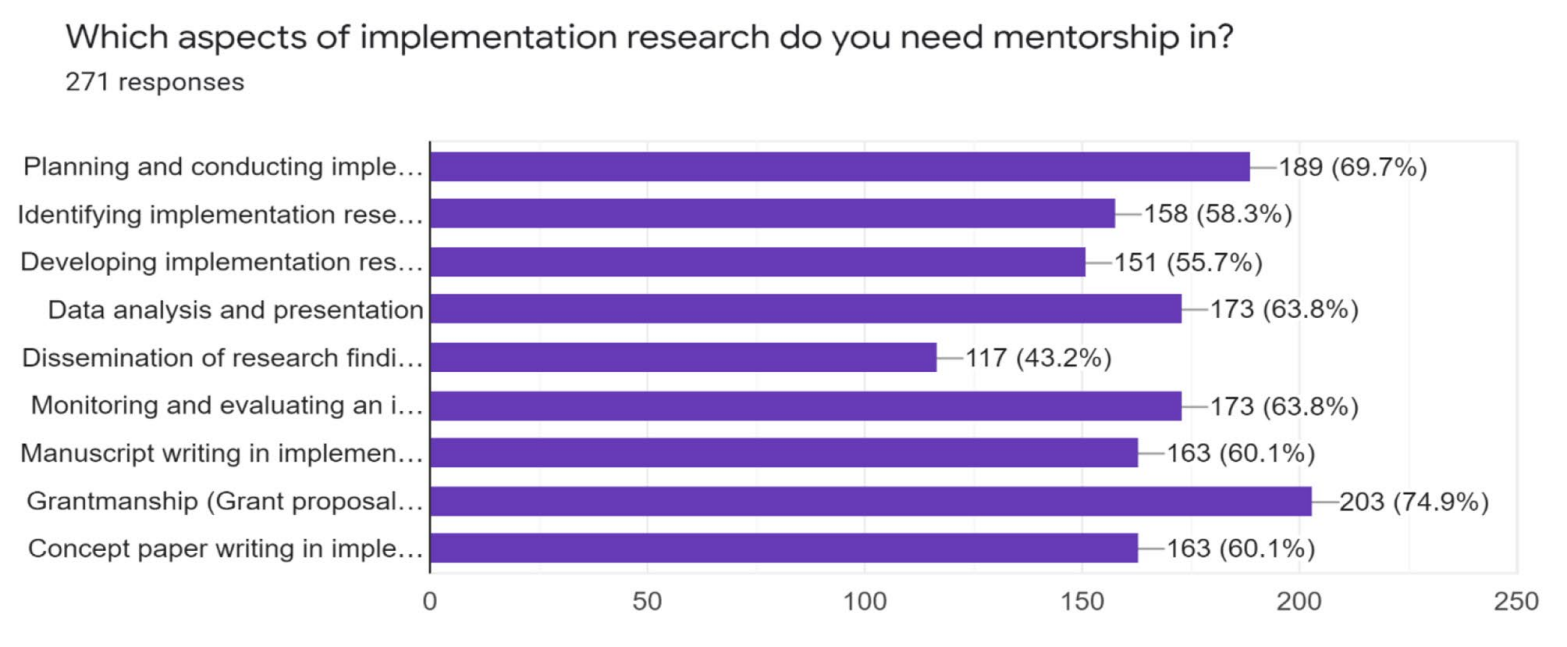
IR capacity building areas

As part of data collection to inform the content of mentorships and strategies. The results as shown in Fig. 3 show that majority, 189 (69.7%) need mentorship in planning and conducting implementation research, 158 (58.3%) need mentorship in identifying implementation research question whilst 203 (74.9%) need mentorship in grantsmanship.

**Fig. 4:**
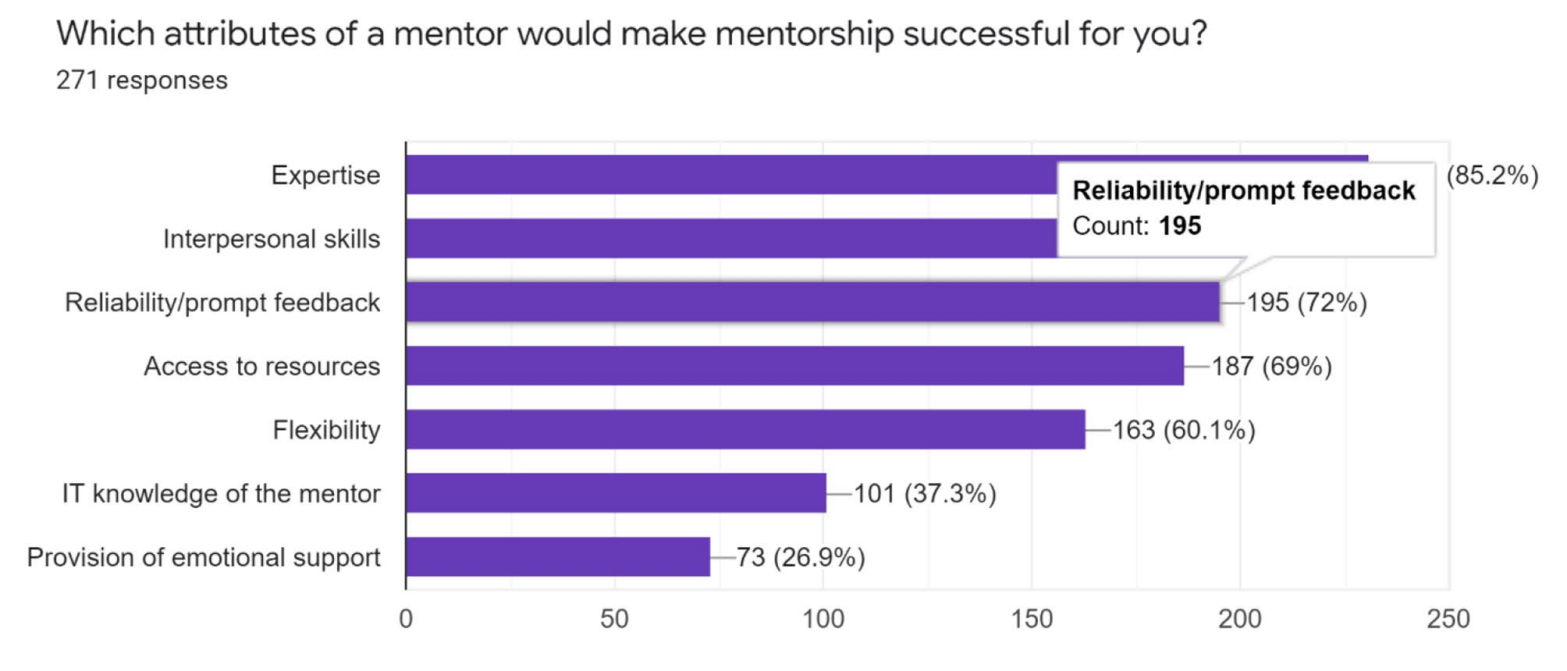
Attributes of a good mentor

In terms of attributes of the mentor, expertise, interpersonal skills, and access to resources were deemed as good qualities of a mentor (Fig. 4).

**Fig. 5:**
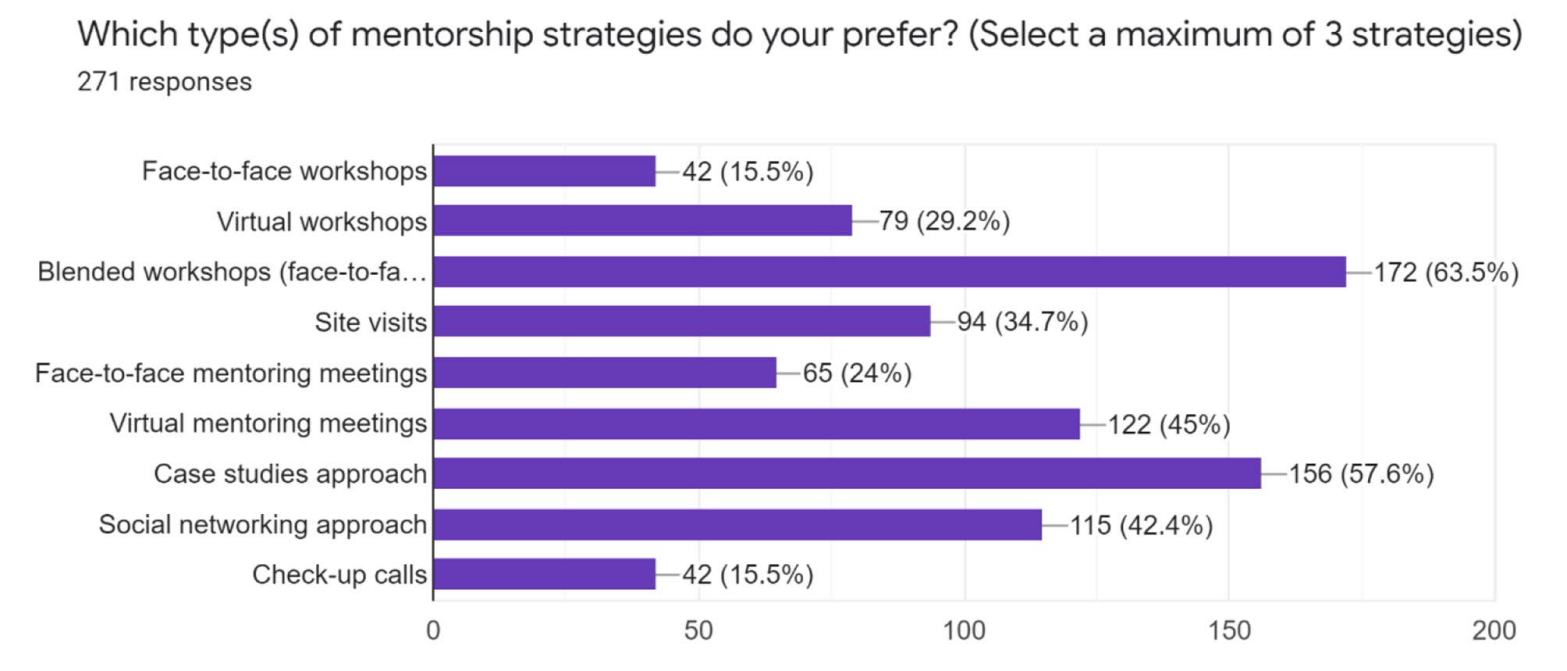
Mentorship strategies

With regards to preferred mentorship strategies as shown in Fig. 5, blended approach was the most preferred. Case studies, virtual mentoring meetings and use of social networking approaches respectively were preferred mentorship strategies.

## Analysis of IR proposals submitted

The proposal went through two review stages. Internally, each submission was reviewed by six mentors and graded. The assessment criteria included: appropriateness of title, relevance of the IR problem, IR Objectives, and feasibility of the methodology. The participants were to identify an implementation challenges and propose interventions to address the health problem in the following disease areas: malaria prevention and control, Buruli ulcer, tuberculosis case detection and adherence to medication, antiretroviral medication, and mass drug administration for lymphatic filariasis (Supplementary File 1). Following internal assessment, the proposals were sent for external review. Each submission was reviewed by three experts in implementation research. An average score was used to determine the winners. The three submissions recommended for award of the research grant have been summarized on Table 3.

**Table 3:** Proposals Recommended for Funding

No.	Title
1.	Alternative implementation strategies of the fourth vaccine dose of the RTS, S in the Cape Coast Metropolis
2.	Factors influencing patients on antiretroviral therapy lost to follow up in Asunafo South District of Ahafo Region, Ghana
3	Assessment of barriers and strategies to tuberculosis treatment adherence in Obuasi Municipal and Obuasi East District: An Implementation Research

### Content of mentorship guide

The results from phases one and two were used to develop and validate a mentorship guide provides insight on mentorship process. The guidance starts by providing working definition of mentorship from practitioners in both academia and policy makers. In addition, the guidance establishes mentor-mentee relationship, the strategies that should be employed and frequency of meeting.

The guide also provides gender dimension and dynamics in mentor-mentee relationship. Whilst acknowledging difference in sex among mentor-mentee as a driver for successful or unsuccessful mentorship, the guide emphasizes the need to situate that within the socio-cultural context. The guidance also articulates participants’ views on the qualities of a good mentor as well as mentee. Some of the qualities of mentors discussed in the mentorship guidance include trustworthy, receptive/approachable, and responsive. A mentee is also expected to be responsive, respectful, innovative and abreast with technology.

In addition, the guide provides a metric for assessing short and long-term goals of the mentorship process. The guide emphasis the need to regularly monitor the activities of both the mentor and mentee. To achieve this, it would require setting of clearly defined deliverables at the beginning of the mentorship process which can be used to measure the outcome of the process as well as establishing good communication. For example, this could include timely submission of a thesis dissertation for a student mentee, the ability of the mentee to publish in peer reviewed journals or secure a research project grant funding.

Furthermore, the mentorship guide also provides guidance on how to end mentorship. The purpose of the mentoring process is to achieve a specific objective. Hence, once the mentorship objective is achieved, the relationship can be weaned off. However, both mentors and mentees can maintain the mentorship relationship for other phases of their lifelong interaction. This was highlighted by both mentees and mentors and incorporated into the mentorship guidance.

## Discussion

Effective mentorship is essential in developing a critical mass of experts in implementation research. Nonetheless the absence of mentorship guidance makes the process ad hoc and not standardized. The study therefore documented the processes in developing a mentorship guidance to help address the challenges in providing mentorship in IR. The findings of the study clearly show that both mentors and mentees agree on the essential role communication plays in the process. The mentorship process involves the building of mutual trust and understanding, that enhances the success of their interaction. As such, mentors can establish and improve the confidence of their mentees by ensuring trust in their communication [26]. Communication between the mentor and mentee is a two-way process involving verbal, and nonverbal sharing of information. According to Freeman, 2016, good communication fosters a positive working relationship and better understanding. Importantly, the mentee feels respected and becomes willing to learn from the mentor [26]. The communication could be verbal and non-verbal, Nonetheless it is important for mentors to remember that they are communicating to mentees when they are speaking and when they are not speaking [27, 28]. It is imperative for the mentor to be conscious of what he or she is communicating nonverbally [26].

Mentorship is an ongoing relationship with series of events governing the interaction between the mentor and the mentee with the ultimate goal of assisting the mentee to become independent in the chosen field [29, 30]. Factors such as socio-cultural and gender norms influence the quality of mentorship that a mentee may gain [30]. As a result, the mentorship guidance emphasizes the need for both mentor-mentee to acknowledge this and develop strategies to mitigate and navigate through these barriers while making the process effective.

Mentoring in High-Income Countries (HIC) is horizontal but strictly hierarchical in LMIC [31]. The absence of a mentorship guide in LMIC settings has the tendency to result in mentors taking advantage of mentees in response to culture or to social norms [32, 33]. The mentorship guidance therefore provides a framework and document to initiate or improve the quality of mentorship in LMICs.

Both mentees and mentors emphasized the need for a mentee to keep a record of all interactions throughout the mentoring process to ensure accountability and continuity [34]. This is relevant to assessing the positive outcomes for both the mentees and the mentors [32]. Previous studies on mentoring indicate that mentored individuals are more productive and have increased knowledge and skills. People who benefit from mentorship practices are more satisfied and committed to their work and exhibit less negative work-related experiences [29, 35, 36]. In a study among 215 primary care research mentees to assess the relation between mentorship and productivity and career development, it was observed that over 66% who had influential mentors conducted research for a longer duration, published more papers, and were more likely to mentor others [37]. A study in Ghana also revealed that teacher trainees who were assigned to mentors had better teaching experience [27]. Given the important role IR plays in addressing health systems implementation challenges, providing guidance on mentorship can help the transfer of knowledge and skills from experienced members of academia and policy implementers to the younger generation of scientist and program implementors.

## Conclusions

The approach for providing comprehensive implementation research training and mentorship for programme has provided an opportunity for both potential mentors and mentees to make inputs into developing a mentorship guidance for LMICs. This guidance would help address the mentorship initiation and implementation challenges.

## Electronic supplementary material

Below is the link to the electronic supplementary material.

Supplementary Material 1

Supplementary Material 2

## Data Availability

The datasets used and/or analysed during the current study are available from the corresponding author on reasonable request on ptabong@ug.edu.gh.

## Abbreviations

ARTC: African Regional Training Centre
IR: Implementation Research
LMIC: Low -and Middle-Income Country
MDR: Multiple Drug Resistant
MOOC: Massive Open Online Course
NTD: Neglected Tropical Disease
NTDP: Neglected Tropical Diseases Programme
PIR: Principles of Implementation Research
SARS-CoV 2: Severe Acute Respiratory Syndrome Coronavirus 2
TB: Tuberculosis
TDR: Tropical Disease Research
