# Supplementary material for: Capacity building through comprehensive implementation research training and mentorship: an approach for translating knowledge into practice
Source: Global Health. 2023 May 25;19:35. doi: 10.1186/s12992-023-00935-8 (PMC10211293; doi:10.1186/s12992-023-00935-8)
Supplement: Supplementary file 1 — Supplementary Material 1 [file 12992_2023_935_MOESM1_ESM.docx]

**SUMMARY OF IR PROSALS**

| **Proposal Number** | **TITLE** | **EXECUTIVE SUMMARY** |
| --- | --- | --- |
| #1 | Motivating Community Drug Distributors Within Districts With High Attrition Rates For Sustainable Mass Drug Administration In The Ahafo Region | Mass Drug Administration (MDA) plays a key role in the supply of Ivermectin which is able to kill microfilaria worms which causes Onchocerciasis also known as River Blindness, the agent is transmitted by Culex Mosquitoes and other mosquito species. Community Drug Distributors (CDDs) plays a significant role in the distribution of Ivermectin to community members, however, they are faced by a lot of challenges including: community level factors, health system associated, and personal level factors. These complexity of factors leads to high CDDs attrition rates which is mostly undocumented and also less examine by many studies, which study will explore and test the effectiveness of potential interventions that will motivate and retain CDDs and sustain the MDA programme. |
| #2 | Assessment Of Barriers And Strategies To Tuberculosis Treatment Adherence In Obuasi Municipal And Obuasi East District: An Implementation Research | Tuberculosis (TB) is an infectious disease caused by *Mycobacterium tuberculosis*. Transmission occurs via droplets from an infected person through coughing and sneezing (World Health Organization, 2020). This disease has plagued humanity for many years and continues to be a major global health problem, with millions of people affected yearly (Santos, 2012; WHO, 2018). Global TB estimates show that 9.9 million people were reported to have developed TB in 2020 with 1.3 million deaths among HIV-negative people and 214,000 deaths among HIV-positive people. Africa is the second highest TB endemic region in the world and contributes to 25% of global TB incidence cases (WHO, 2021). In Ghana, the second national TB survey revealed a national prevalence rate of 290 per 100,000 population. This rate is about three times higher than the estimated 92 per 100,000 by WHO (Bonsu et al., 2014). The World Health Organization (WHO) recommends Stop TB Strategy based on Directly Observed Therapy Short-course (DOTS) to control TB (WHO, 2003). The DOTS strategy aims to ensure that TB patients take a standard short-course of chemotherapy under guided supervision to cure the disease and prevent its spread. TB patients obtain assistance through their treatment duration and are encouraged to complete treatment in order to prevent resistance to any of the available anti-tuberculosis drugs (WHO, 2003). The DOTS strategy was initially hospital based where TB patients were admitted and observed to take anti-TB drugs by a hospital staff. However, community-based therapy was proven effective and accepted by the WHO as a preferable intervention to enhance successful TB treatment. This was due to challenges arising from the hospital-based activity, including inadequate hospital space capacity to admit all TB patients. This Community-based intervention (CBI) was designed to aid the National TB control programmes to increase case detection, cure the patients, interrupt transmission of the TB bacilli to other people, and prevent drug resistance (WHO, 2018; Zenebaba, Bonsa & Sahiledengle, 2021). In this context, treatment monitoring and assistance are to be offered by a treatment supporter either by a community volunteer, lay health worker or preferably a family member at the community level (Arshad et al., 2014).  Despite the effectiveness of the community-based DOTS strategy, adherence to treatment is a major challenge in many countries, given the complexity, modest tolerability, and long duration of the currently available treatment regimen for both drug-susceptible and resistant TB (Alipanah et al., 2018). Low adherence to treatment resulting from discontinuity of treatment leads to adverse outcomes including lost to follow-up, treatment failure, deaths and resistance to the TB drugs (Zenebaba et al., 2021). These adverse treatment outcomes have direct influence on the first pillar of the End TB Strategy of the WHO which denotes Integrated, Patient Centered Care and Prevention – and necessitate “treatment of all people with TB including drug-resistant TB; and patient support” as this may not be achieved if the problem of low treatment adherence is not addressed (WHO, 2014).  In Ghana, despite substantial improvements, TB treatment success rate remains below the 90% target set out in the country’s 2015-2020 strategic plan (Bonsu et al., 2014). The magnitude of problem of adverse treatment outcomes especially lost to follow-up and TB deaths varies across districts within the country. Usually, in districts which are highly burdened with TB lost to follow-up and death rate are unacceptably high. In Ashanti region for instance, a lot of TB patients in the two highly burdened TB districts in Obuasi (i.e. both Obuasi Municipal and Obuasi East district) are mostly either lost to follow-up or dead. This situation has been consistently high over the past decade. Recently in 2021, 72 (30.4%) of 237 TB patient enrolled in TB care in both districts in Obuasi experienced adverse treatment outcomes with 55 (23.2%) lost to follow-up and 17 (7.2%) TB deaths (District Annual TB Report, 2021).  Although some studies in different areas across the globe have identified some implementation gaps resulting in low TB treatment adherence (Thiam et al., 2007; Ferreira et al., 2011; Ritchie et al., 2020), to the best of our literature search, there is no evidence of studies conducted in Ashanti region and in districts in Obuasi in this regard. This study thus seeks to explore the main barriers to TB treatment adherence from both patient and healthcare worker’s perspective, formulate potential patient-centered strategies, and evaluate the potential impact of the strategies on improving TB treatment outcome in the two districts in Obuasi. |
| #3 | Improving TB case detection using WHO Symptom-Based Screening Tool in West Mamprusi Municipality of The North East Region | Tuberculosis is an old disease but one which all health workers around the world are finding difficult to control. The World Health Organization in 2003 declared TB a global emergency on recognition of the growing importance of TB as a public health problem. About one-third of the world’s population is infected with Mycobacterium tuberculosis. Tuberculosis kills more than any other infectious disease agent3. According to the Global Tuberculosis Report in 2019, TB is considered the ninth cause of death worldwide and the leading cause of mortality by a single infectious agent, with the highest rate of infections and death toll mostly concentrated in developing, low and middle-income countries1. Deaths from TB account for 25% of all avoidable deaths in developing countries3. In 2018 alone, there were 1.5 million deaths and 10 million new cases globally, among whom half a million had rifampicin-resistant TB. The annual rate of decline in TB incidence is still much lower than what would be needed to end the TB epidemic by 20304.  Tuberculosis (TB) is an infectious disease caused by the bacterium *Mycobacterium tuberculosis* (MTB), and it is a persistent public health problem globally. TB is spread from one person to another principally by airborne transmission. In a small proportion of cases, the bacillus is transmitted to humans from infected cows through drinking non-sterilized milk (*Bovine TB*). This mode of transmission plays only a minor role in the natural history of the disease in humans3. TB can affect any organ in the body but Pulmonary tuberculosis is the most frequent type of TB and it is infectious. Extrapulmonary TB is less frequent and non-infectious2. The main reservoir of M. tuberculosis is the patient with pulmonary tuberculosis. Such patients may have pulmonary cavities in the lungs that contain several bacilli and when they cough, they release the bacilli into the atmosphere. When a susceptible host (human) breath in the pathogen, infection occurs. People who are infected may not develop the disease based on the host immune factors. Mostly TB infection develops into full-blown disease in immunocompromised people such as People Living with HIV/AIDS (PLHIV). TB is an opportunistic infection among PLHIV and it is the leading cause of death among this group of people3.  In Ghana, TB remain a major public health problem despite increased efforts in prevention and control measures, as well as the availability of effective short-course tuberculosis treatment, the incidence of TB is still on the rise5. Poor TB case detection leads to increased transmission and high TB prevalence rates, as each active case has the capacity to infect 10–15 people per year9. |
| #4 | Implementation Of Community-Based Buruli Ulcer Case Search And Management In The Hohoe Municipality (Volta Region) 2022. | Buruli ulcer is a devastating neglected tropical illness that typically appears as non-ulcerated lesions and if left untreated, it can progress to major skin ulcerations, joint, and bone degradation. Aside the long-lasting health impact of Buruli ulcer (BU) such as limb amputations, BU also imposes an economic cost on sufferers and their families including an indirect cost treatment estimated at $550 in addition to 265 productive days lost among ulcerative cases of BU. Buruli ulcer is under-reported in endemic countries, according to cross-sectional surveys, for a variety of reasons, including the disease's chronic, stigmatizing nature, its rural distribution, patients' poor access to health care or preference for traditional healers, and a lack of awareness or resources within health systems. Buruli Ulcer case discovery in the Hohoe municipal has not been consistent. Over the past years, discovered cases of Buruli Ulcer in the Hohoe municipal was 2018(26), 2019(12), 2020(2) and 2021(0). This presents the challenges of low Buruli ulcer case detection in the Hohoe municipal and in most cases, the condition is detected when it is in stage III as a result of late reporting and low case detection. The aim of this research is to assess the viability of equipping community-based volunteers, Nurses and clinicians as supporting Buruli Ulcer health surveillance personnel to conduct community-based active surveillance for early Buruli ulcer case detection in the Hohoe municipal of the Volta region of Ghana.  This research will be an epidemiologic study designed to test and evaluate a community surveillance response system in Hohoe for a period of 12 months. It will be a longitudinal study with baseline and end line surveys to compare pre- and post- implementation perceptions among the population. Ten Community-Based Volunteers, ten Community Health Officers, five Disease Control Officers and ten selected clinical staff will be trained by the Ghana Health Service for the surveillance-response, diagnosis and treatment of Buruli ulcer. The surveillance-response system will be established and implemented from April, 2022 to March, 2023. Thus, once every 12 monthly rounds will be led by community-based volunteers in collaboration with local health workers. Pre and post intervention data will be collected from the participating community to assess the efficacy of the BU surveillance process. Data will be analyzed with Epi info 7 and the results of the findings will be presented in tables and figures with levels of significance considered at p<0.05.  This research will enhance early case detection, diagnosis and treatment. The surveillance- response system implemented will not only be able to pick cases at early stages to eliminate or, at least, reduce severe and debilitating ulcers associated with late reporting at health facilities, which causes morbidity and disabilities, but also help to rediscovered old cases that are hidden from the view of the health system. Furthermore, this project will seek to train clinicians in the various modalities of BU diagnosis to confirm BU cases and to provide treatment options based on the clinical presentations. |
| #5 | Alternative Implementation Strategies For The Administration Of The Fourth Vaccine Dose Of The Rts, S In The Cape Coast Metropolis. | Malaria is an endemic disease in Ghana, and the entire population is at risk of contracting it. Numerous malaria control initiatives are being implemented in Sub-Saharan Africa. For example, in Ghana, interventions such as targeted indoor residual spraying (IRS), insecticide-treated nets (ITNs), intermittent preventive treatment for pregnant women (IPTp), malaria monitoring, and malaria chemotherapy are used. To facilitate the malaria eradication project, efforts were put in place to develop a malaria vaccine. As a result, the RTS, S recombinant protein-based vaccine was approved by European authorities in July 2015, after two decades of clinical trials. This was a significant step forward in vaccine development. Despite the remarkable achievements in RTS, S uptake, there is increasing concern about receiving the fourth vaccine dose across the country's implementation centres. Therefore, this study aims to identify possible implementation strategy approaches that would enhance adherence significantly to improve the uptake level for the fourth dose of the Malaria RTS, S vaccine. The study will employ the concurrent exploratory research design of the mixed-method research strategy. Mixed methods present opportunities to combine qualitative interviews with the computer-assisted questionnaire administration to seek convergence and corroboration of findings from different methods. The researcher intends to conduct the study in the Cape Coast Metropolis with a sample size of 643 mothers with children age 2-3 years. For the qualitative part of the study, a focused group discussion will be organised among mothers with the five sub metro. Further, in-depth interviews will be conducted among defaulter mothers. Communinity health nurses will also be interviewed. Thematic analysis will be used to analysed the qualitative data. Ordinal logistic regression will be modelled on the quantitative dataset. |
| #6 | Factors Influencing Patients On Antiretroviral Therapy Lost To Follow Up In Asunafo South District Of Ahafo Region, Ghana | Even though there has been an improved and highly successful program coverage with ART, significant numbers of adults and children drop out of care at various points along their treatment pathway, hence, treatment gains fail to reach sufficient numbers of these populations. In sub-Saharan Africa, it has been shown that about half of the people who test HIV positive are lost between testing and being assessed for eligibility for therapy. The objective of this study is to identify the factors influencing patients lost to follow up on ART in HIV care clinics in Asunafo South District of Ahafo Region. The outcome of this study is expected to deepen stakeholders’ understanding of the challenges ART patients and HIV clinic staff face in continuing on ART, and ensuring effective contact tracing or reengagement of ART clients in Ghana and to identify measures with the potential to improving retention in care and outcomes for PLHIV in Ghana. |
