# Supplementary material for: Capacity building through comprehensive implementation research training and mentorship: an approach for translating knowledge into practice
Source: Global Health. 2023 May 25;19:35. doi: 10.1186/s12992-023-00935-8 (PMC10211293; doi:10.1186/s12992-023-00935-8)
Supplement: Supplementary file 2 — Supplementary Material 2 [file 12992_2023_935_MOESM2_ESM.docx]

Dear Editor-in-chief,

**RESUBMISSION OF MANUSCRIPT: CAPACITY BUILDING THROUGH COMPREHENSIVE IMPLEMENTATION RESEARCH TRAINING AND MENTORSHIP: AN APPROACH FOR TRANSLATING KNOWLEDGE INTO PRACTICE**

I wish on behalf of co-authors to resubmit to you a manuscript titled “Capacity Building through Comprehensive Implementation Research Training and Mentorship: An approach for translating knowledge into practice” to your journal for consideration and possible publication.

We have addressed all the comments by the reviewers and provided a point-by-point response to the reviewers comments.

This manuscript has not been published in any journal or is under consideration in any journal. Any future correspondence should be addressed to me.

I look forward to receiving any comments.

Thank you.

Yours faithfully,

Philip Teg-Nefaah Tabong (PhD)

(Corresponding Author)
